# Efficacy of Silexan in subthreshold anxiety: meta-analysis of randomised, placebo-controlled trials

**DOI:** 10.1007/s00406-017-0852-4

**Published:** 2017-11-17

**Authors:** Hans-Jürgen Möller, Hans-Peter Volz, Angelika Dienel, Sandra Schläfke, Siegfried Kasper

**Affiliations:** 1 Clinic and Polyclinic for Psychiatry and Psychotherapy, Ludwig Maximilian University, Munich, Germany; 2 Hospital for Psychiatry, Psychotherapy and Psychosomatic Medicine Schloss Werneck, Werneck, Germany; 3 Department of Clinical Research 1, Dr. Willmar Schwabe GmbH & Co. KG, Karlsruhe, Germany; 4 Department of Biostatistics, Dr. Willmar Schwabe GmbH & Co. KG, Karlsruhe, Germany; 5 Department of Psychiatry and Psychotherapy, Medical University of Vienna, Währinger Gürtel 18-20, 1090 Vienna, Austria

**Keywords:** Silexan, Lavender oil, Subthreshold anxiety, Treatment efficacy, Meta-analysis

## Abstract

Subthreshold psychiatric disorders do not fully meet the diagnostic criteria of syndromal disorders but may be associated with comparable disability. To investigate the anxiolytic effect of Silexan, an active substance from lavender oil for oral administration, in patients with subthreshold anxiety, a meta-analysis that included all published trials with Silexan in this indication was performed. Three randomised, placebo-controlled trials in subthreshold anxiety disorders (anxiety disorder not otherwise specified, restlessness and agitation, mixed anxiety and depressive disorder) were included. Eligible participants with a baseline Hamilton Anxiety Rating Scale (HAMA) total score ≥ 18 points received 1 × 80 mg/day Silexan or placebo for 10 weeks. Outcomes included the HAMA, the Pittsburgh Sleep Quality Index, the Zung Self-rating Anxiety Scale, the Clinical Global Impressions questionnaire and the SF-36 health status inventory. Data were analysed using meta-analysis based on pooled raw data of individual patients (random effects models). A total of 697 patients were assessed for efficacy. Silexan was superior to placebo in reducing the HAMA total score during 10 weeks’ treatment [mean value difference, 95% confidence interval: 3.83 (1.28; 6.37) points]. Superiority was comparably pronounced for psychic and somatic anxiety as well as for observer- and self-rated anxiety. Silexan had a beneficial effect on sleep (secondary to the anxiolytic effect) without causing sedation and improved the patients’ health-related quality of life. Adverse event incidence in both treatment groups was comparable [risk ratio: 1.06 (0.85; 1.33)]. Silexan has a significant and clinically meaningful anxiolytic effect in subthreshold anxiety. The results cannot be generalised to other lavender oil products.

## Introduction

Subthreshold anxiety disorder (SSAD, subsyndromal anxiety disorder) is a very common but nevertheless often underdiagnosed and undertreated condition that refers to individuals with clinically relevant symptoms of anxiety who meet several, but not all diagnostic criteria of ‘syndromal’ generalised anxiety disorder (GAD) according to the standards of the Diagnostic and Statistical Manual of Mental Disorders (DSM) of the American Psychiatric Association or of the WHO’s International Classification of Diseases (ICD) [1]. It is in fact so poorly recognised that the current versions of the applicable classification systems do not even offer a specific diagnostic category; instead, clinicians have to code SSAD as Other Specified Anxiety Disorder (300.09) or Unspecified Anxiety Disorder (300.00) in DSM-5 or as Anxiety Disorder, Unspecified (F41.9) or Mixed Anxiety and Depression (F 41.2; in case of additional subthreshold but clinically relevant depressive symptoms) in ICD-10.

Epidemiological data suggest that the population prevalence of SSAD may exceed that of GAD in Europe and North America by a factor of about three [1–3]. Moreover, a recent study showed that nearly three out of four adult individuals seeking treatment for clinically relevant worry failed to meet the criteria for ‘syndromal’ GAD only by a single criterion [4]. There is almost uniform agreement between researchers that SSAD causes relevant functional impairment and suffering, has a detrimental effect on quality of life, is associated with a high level of co-morbidity and bears a considerable risk of exacerbation to threshold anxiety, mood disorder or substance use disorders [1, 5, 6]. Moreover, SSAD, in contrast to GAD, was found to be associated with a significant decrease in perceived social support [7]. These findings underline the need for an early and adequate treatment of SSAD, which could also efficiently and cost-effectively prevent a progression to the syndromal level [8, 9].

Silexan, which has been licensed in 14 countries worldwide and is the active substance of a medicinal product marketed in Germany, is produced from lavender and causes a potent inhibition of voltage dependent calcium channels (VOCCs) in synaptosomes, primary hippocampal neurons and stably overexpressing cell lines [10], which have been shown to play an important role in both anxiety and depression. Inhibition of VOCCs can lead to an attenuation of the overreaching, situationally inadequate stress response of the central nervous system associated with anxiety and mood disorders [11]. Moreover, Silexan significantly reduces the 5-HT_1A_ binding potential in the brain clusters encompassing the temporal gyrus, the fusiform gyrus, the hippocampus, the insula and the anterior cingulate cortex, which may lead to an increase of extracellular serotonin levels [12].

In randomised, double-blind, placebo-controlled clinical trials Silexan showed a pronounced anxiolytic effect in threshold GAD and also in indications having subthreshold anxiety as a principal feature (SSAD, mixed anxiety and depressive disorder (MADD), anxiety-related restlessness and agitation) [13–16]. In order to obtain a comprehensive overview of the anxiolytic efficacy of Silexan in subthreshold anxiety, we performed a meta-analysis of randomised, placebo-controlled clinical trials that investigated the effect of the active substance in these indications.

## Methods

### Searches, study and participant characteristics

For our meta-analysis individual patient data of three double-blind, randomised, placebo-controlled, multicentre phase III clinical trials in patients with subthreshold anxiety [14–16] were obtained from the manufacturer of Silexan. To identify any additional studies performed with Silexan in patients with subthreshold anxiety, we performed free-text searches of all fields of the MEDLINE database as well as of the ClinicalTrials.gov registry, the EMA Clinical Trials Register, and the ISRCTN registry for any records that included the search term ‘Silexan’ in combination with ‘anxiety’ and had been entered before 31 March 2017 (no further restrictions applied). In addition to the original publications presenting the results of the three trials above [14–16], the MEDLINE search retrieved a total of 14 other publications. Four described randomised, controlled trials with Silexan in other psychiatric indications [13, 17–19], and three covered non-clinical studies with Silexan [10, 12, 20]. Retrieved publications also included a contraceptives interaction study [21] and a case report [22]. The remaining five publications were review articles from which no additional studies with Silexan in patients with subthreshold anxiety could be identified [14, 23–26]. Searches performed in clinical trials registries also did not reveal any additional studies with Silexan performed in the indication of interest. The search results thus indicate that the three randomised, controlled trials performed in patients with psychiatric diagnoses characterised by subthreshold anxiety appear to be the only randomised, controlled studies performed with Silexan in the population of interest by the time when our searches were completed. The remaining identified trials covered different indications and were thus excluded.

Among the eligible trials, Study A assessed the efficacy of Silexan patients with SSAD (classified as ‘anxiety disorder not otherwise specified’ according to the DSM-IV 300.00 and as ‘anxiety disorder, unspecified’ according to ICD-10 F41.9) [14]. The participants of Study B suffered from restlessness and agitation (ICD-10 R45.1) as well as from disturbed sleep [16]. Study C was performed in patients diagnosed to be suffering from MADD (ICD-10 F41.2) [15].

The main characteristics and criteria for participant selection of the included trials are summarised in Table 1. The participants of all studies were female or male, adult out-patients who consulted a general practitioner or a psychiatrist. Each trial started with a 3–7 days qualification phase after which eligible patients were randomised and received Silexan or placebo for a scheduled period of 10 weeks, with assessments performed at baseline (randomisation) and at the end of weeks two, four, six, eight, and ten (Studies A and B), or at the end of weeks one, two, four, seven, and ten (Study C). For eligibility, patients of all trials had to meet the diagnostic criteria for the condition under investigation and had to present with a total score ≥ 18 points for the Hamilton Anxiety Rating Scale (HAMA) as well as with minimum scores for two individual HAMA items as shown in Table 1 at both inclusion and baseline.

**Table 1:** Main study characteristics and participant selection criteria

Trial	A [14]	B [16]	C [15]
Design characteristics	Double-blind, randomised, placebo-controlled, Multicentre		
Diagnosis for inclusion	Anxiety disorder not otherwise specified (DSM-IV 300.00; ICD-10 F41.9)	Restlessness and agitation (ICD-10 R45.1)	Mixed anxiety and depressive disorder (ICD-10 F41.2)
Anxiety specific inclusion criteria	HAMA total score ≥ 18 points; HAMA items ‘Anxious mood’ and ‘Insomnia’ ≥ 2 points	HAMA total score ≥ 18 points; HAMA items ‘Tension’ and ‘Insomnia’ ≥ 2 points	HAMA total score ≥ 18 points; HAMA items ‘Anxious mood’ and ‘Depressed mood’ ≥ 2 points
Interventions	1 × 80 mg/day Silexan or placebo, 10 weeks		
Primary efficacy outcome measure, anxiety	HAMA total score change, baseline—end of treatment		
Further efficacy outcome measures	SAS; PSQI; SF-36; CGI	SAS; PSQI; CGI	HADS; SF-36; CGI

*HAMA* Hamilton Anxiety Rating Scale, *SAS* Zung Self-rating Anxiety Scale, *HADS* Hospital Anxiety and Depression Scale, *PSQI* Pittsburgh Sleep Quality Index, *SF-36* SF-36 Health Survey Questionnaire, *CGI* clinical global impressions

### Ethical conduct

All trials included into our meta-analysis were performed following the principles of Good Clinical Practice and the Declaration of Helsinki after obtaining approval from an Independent ethics committee. The studies were registered in the EudraCT database (all studies) as well as in the ISRCTN registry (studies A and C). All participants provided written informed consent.

### Interventions

Silexan is a special active substance with an essential oil produced from *Lavandula angustifolia* flowers by steam distillation that complies with the monograph Lavender oil of the European Pharmacopoeia and exceeds the quality requirements of the monograph. Batch to batch consistency is assured by a well-defined, standardised manufacturing process. Immediate release soft gelatine capsules containing 80 mg of Silexan or identically matched placebo capsules were used. The smell of the investigational treatments was matched by flavouring the capsules containing placebo with 1/1000 of the amount of lavender oil contained in the Silexan capsules. In all studies randomised patients received Silexan monotherapy and had to administer one capsule per day unchewed in the morning. The daily dose was chosen in accordance with recommended dose of the marketed product.

### Meta-analysis outcomes

The anxiolytic effect of Silexan was assessed by analysing HAMA total score change between baseline and treatment end, which was the pre-defined main outcome in all eligible studies. Moreover, we analysed the change of the HAMA psychic and somatic anxiety subscores [27] as well as of the individual item scores for anxious mood, tension, and sleep. Anxiety self-ratings were included into the meta-analysis using the change of the total score of the Zung Self-rating Anxiety Scale (SAS; studies A and B) or of the anxiety rating of the Hospital Anxiety and Depression Scale (HADS; study C). Sleep quality was assessed by analysing the total score change of the Pittsburgh Sleep Quality Index (PSQI; studies A and B only). Meta-analyses were also performed for the changes of the mental health and the physical health subscores of the SF-36 Health Survey Questionnaire which assesses health-related quality of life (studies A and C only), and of Items 1 and 2 of the Clinical Global Impressions scale (CGI).

Our meta-analysis also included assessments of treatment response and remission. We defined response as a decrease of the HAMA total score by at least 50% of the baseline value or as a score equal to or less than two points for CGI item 2 (i.e., much or very much improved compared to project admission), both assessed at treatment end. Remission was defined as a HAMA total score of less than ten points or of less than or equal to seven points at treatment end.

In all trials tolerability and safety were primarily assessed by monitoring adverse events (AEs).

### Bias assessment

Bias assessment on the study level was performed by an independent assessor who was not involved in the planning, conduct, analysis or interpretation of any of the eligible trials, using the Cochrane Collaboration’s tool for assessing risk of bias [28]. Assessments were based upon the applicable publications, the patient raw data, and on the original protocols and the full integrated study reports made available to the authors and to the assessor.

### Statistical methods

The meta-analysis of treatment efficacy was based on the original (raw) data of the included trials and was performed for the primary efficacy analysis data sets (full analysis set, FAS) of the original protocols. For comparability with the published results of the trials, missing data for efficacy outcomes were imputed by carrying forward the last valid observation.

Patient age, sex, and premature withdrawal rate were analysed using descriptive statistics. Within each trial continuous outcomes were analysed using analysis of covariance (ANCOVA) with treatment as a factor, the intraindividual difference between treatment end and baseline for the outcome of interest as the dependent variable, and the baseline value of the outcome as a covariate. Rating scales were analysed as continuous outcomes. For the analysis of CGI item 2 (‘Change from project admission’), which inherently includes change from baseline and which was thus assessed only at post-baseline visits, the baseline value of CGI item 1 (‘Severity of illness’) was used as a covariate.

Meta-analysis methods were pre-defined in a statistical analysis plan. We used a two-stage individual participant data (IPD) meta-analysis approach according to which the outcomes of interest were first analysed equally within each study and then combined using ‘traditional’ meta-analysis [29, 30]. For continuous outcomes, we used the marginal (adjusted) mean values and their estimated standard deviations as input for the meta-analysis and computed random effects models based on the treatment group mean value difference. Inverse variance weighting was used for combining the results of the single trials. We applied the DerSimonian–Laird method to calculate the variance between the trials. In the meta-analysis of self-rated anxiety, which combined ratings originating from the SAS and the HADS clinical questionnaires, the bias corrected Hedges’ G was calculated as an estimate for the combined, standardised mean value difference between the treatments in order to account for the different scales. For the HAMA total score difference and the clinical global impression of change from baseline (CGI item 2) the bias corrected Hedges’ G was calculated in addition in order to facilitate the comparison of results with other published work. Meta-analyses of binary outcomes (response, remission, and AE rates) were based on relative risk. For response and remission, random effects models were used, and trial results were combined according to the inverse variance method. Meta-analyses AE rates were performed using fixed effects models with Mantel–Haenszel weighting for combining the trial results. Additionally meta-analyses of binary outcomes based on risk differences were performed to calculate numbers needed to treat and numbers needed to harm.

For all analyses two-sided *p* values ≤ 0.05 were considered descriptively significant.

Heterogeneity between the trials was assessed using the *I*^2^ statistic in accordance with the criteria proposed in section 9.5.2 of the Cochrane Handbook for Systematic Reviews of Interventions [31].

Meta-analyses were computed with the R software package meta (version 4.3.2) using functions metacont and metabin for continuous and binary data, respectively. All other analyses were performed in SAS statistical software version 9.3.

## Results

### Study and participant characteristics

Risk of within-study bias assessments are provided in Table 2. All eligible studies [14–16] were conceived by the same working group and were performed according to very similar protocols except for the diagnosis for inclusion. Elevated risk of bias related to incomplete outcome data in study B [16] was attributable to a somewhat higher premature withdrawal rate for lack of efficacy in the placebo group (5/84 patients compared to 3/86 for Silexan) and a higher rate of withdrawals for adverse events in the Silexan group (4/86 versus none for placebo), in combination with last observation carried forward missing data imputation.

**Table 2:** Risk of bias assessments according to Higgins et al. [28]

Trial	A [14]	B [16]	C [15]
Random sequence generation	Low	Low	Low
Allocation concealment	Low	Low	Low
Blinding of participants and personnel	Low	Low	Low
Blinding of outcome assessment	Low	Low	Low
Incomplete outcome data	Low	High^a^	Low
Selective reporting	Low	Low	Low
Other sources of bias	Low	Low	Low

^a^Probably favouring placebo

Across all trials included into our analyses 709 patients (Silexan 356; placebo 353) were randomised, 704 (353 and 351) were assessed for safety, and 697 (349 and 348) were analysed for efficacy. Pooled premature withdrawal rates were 12.6 and 10.5% for Silexan and placebo, respectively.

Demographic characteristics and baseline values of efficacy outcomes are shown in Table 3. More than two-thirds of the participants of all studies were female. Across all trials, the patients in both treatment groups were on average about 47 years old.

**Table 3:** Baseline characteristics [*n*, %, or mean (SD)]

Study	A [14]		B [16]		C [15]	
Treatment	Silexan	Placebo	Silexan	Placebo	Silexan	Placebo
Safety analysis set	107	109	86	84	160	158
Full analysis set	104	108	86	84	159	156
% Female	73.1	76.9	72.1	71.4	66.0	72.4
Age (years)	45.6 (11.4)	46.6 (11.3)	48.0 (11.3)	46.9 (12.7)	47.7 (12.6)	47.9 (12.6)
HAMA total score	26.8 (5.4)	27.1 (5.2)	25.5 (6.0)	26.5 (6.1)	25.7 (5.6)	25.7 (5.2)
Anxiety self-rating^a^	60.1 (9.9)	61.1 (10.1)	54.5 (12.3)	55.9 (10.3)	12.7 (3.6)	12.4 (3.5)
CGI severity of illness	4.6 (0.7)	4.7 (0.6)	4.4 (0.7)	4.5 (0.7)	4.4 (0.7)	4.4 (0.7)
PSQI total score	12.3 (2.9)	12.5 (3.0)	12.2 (205)	12.7 (2.7)	NA	NA
SF-36 physical health	51.7 (21.7)	53.2 (22.1)	NA	NA	48.2 (23.5)	49.3 (22.7)
SF-36 mental health	32.3 (17.4)	32.6 (21.2)	NA	NA	30.0 (19.5)	33.4 (21.3)

All results apply to the full analysis set. SF-36: higher values indicate less symptom severity; for all other scales, lower values indicate less symptom severity

*NA* not assessed, *HAMA* Hamilton Anxiety Rating Scale, *CGI* Clinical Global Impressions, *PSQI* Pittsburgh Sleep Quality Index, *SF-36* SF-36 Health Survey Questionnaire

^a^Trials A, B: Zung Self-rating Anxiety Scale (SAS) total score; trial C: Hospital Anxiety and Depression Scale (HADS), anxiety subscale

Within-trial baseline mean value differences for the HAMA total score did not exceed one point, and the mean value differences for HAMA ‘core’ items Anxious mood, Tension, and insomnia never exceeded 0.1 points (data not shown). The baseline values of the remaining efficacy outcomes presented in Table 3 also indicate that the treatment groups within the included trials were essentially comparable at baseline.

### Anxiolytic effect

**Fig. 1:**
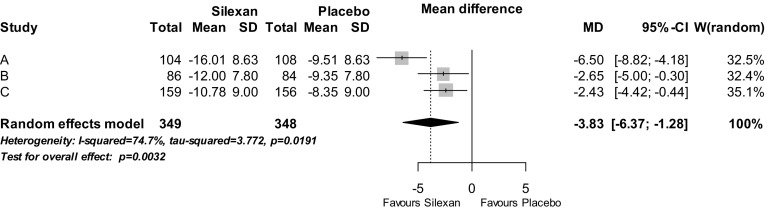
Hamilton Anxiety Rating Scale total score—change between baseline and treatment end (*SD* standard deviation, *MD* mean value difference, *CI* confidence interval, *W* weight)

The main results for the studies’ pre-defined primary or co-primary outcome measure, HAMA total score change between baseline and treatment end, are presented in Fig. 1. In the meta-analysis Silexan was significantly superior to placebo. The figure also shows that Silexan was superior to placebo in each of the three included trials, with the largest treatment effect in trial A. The standardised mean value difference (Hedges’ G) was 0.45 (95% CI 0.15; 0.74, *p* = 0.003).

Table 4 shows that the items conventionally assigned to the psychic (items 1–6, 14) and somatic anxiety subscores of the HAMA (items 7–13) contributed comparably to the over-all effect observed for the total score. Significant advantages for Silexan over placebo were also observed in the meta-analyses of items Anxious mood, Tension, and Insomnia (other items were not analysed separately). For all analyses of HAMA scores and items heterogeneity (ranging between *I*^2^ = 0% and *I*^2^ = 78.5%) was caused by between-trial differences regarding treatment effect size whereas the direction of the treatment group differences always favoured Silexan.

**Table 4:** Hamilton Anxiety Rating Scale, subscores and selected items-meta-analysis results for change between baseline and treatment end (Silexan—placebo; pooled random effect)

Outcome	*N* (Silexan/placebo)	*I*^2^ (%)	Mean value difference^a^ (95% confidence interval)	*p*
Somatic anxiety (subscore)	349/348	68.1	− 1.70 (− 2.77; − 0.63)	0.002
Psychic anxiety (subscore)	349/348	74.5	− 2.15 (− 3.66; − 0.65)	0.005
Anxious mood (item)	349/348	50.2	− 0.43 (− 0.65; − 0.21)	< 0.001
Tension (item)	349/348	0.0	− 0.38 (− 0.54; − 0.22)	< 0.001
Insomnia (item)	349/348	78.5	− 0.38 (0.73; − 0.03)	0.034

^a^Negative values favour Silexan

**Fig. 2:**
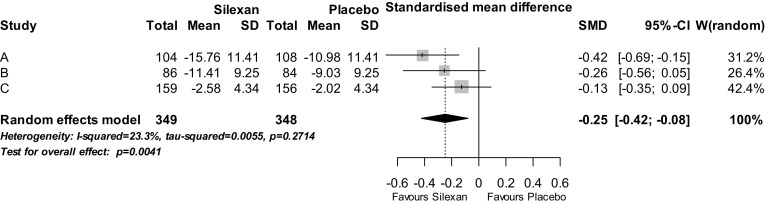
Anxiety self-rating (studies A and B: Zung Anxiety Self-rating Scale total score; study C: Hospital Anxiety and Depression Scale, anxiety subscore)—change between baseline and treatment end (*SD* standard deviation, *SMD* standardised mean value difference, *CI* confidence interval, *W* weight)

The results for the observer-rated HAMA were supported by the study participants’ anxiety self-ratings obtained with the Zung SAS (trials A and B) and the HADS (trial C; Fig. 2), the values of which cannot be compared directly due to the different ranges of the 2 self-rating scales. The meta-analysis model again showed a significant treatment group difference (*p* = 0.004) favouring Silexan while only minor heterogeneity was observed.

### Over-all clinical condition

In the included trials the patients’ over-all severity of impairment rated with CGI item 1 decreased by mean values between 1.14 and 2.06 points for Silexan and by between 0.66 and 1.19 points for placebo. The meta-analysis showed a significant over-all effect favouring Silexan (Table 5). Heterogeneity was again caused by differences regarding the magnitude, not the direction of the treatment effect.

**Table 5:** Additional efficacy outcomes—meta-analysis results for change between baseline and treatment end (Silexan—placebo; pooled random effect)

Outcome	*N* (Silexan/placebo)	*I*^2^ (%)	Mean value difference (95% confidence interval)	*p*
CGI item 1 (severity of impairment)^a^	335/337	63.8	− 0.54 (− 0.85; − 0.23)	< 0.001
PSQI total score^a^	190/192	26.9	− 1.36 (− 2.28; − 0.44)	0.004
SF-36 physical health subscore^b^	252/256	0.0	7.32 (3.88; 10.77)	< 0.001
SF-36 mental health subscore^b^	252/256	16.0	10.19 (5.78; 14.61)	< 0.001

*CGI* Clinical Global Impressions, *PSQI* Pittsburgh Sleep Quality Index, *SF-36* SF-36 Health Survey Questionnaire

^a^Negative values favour Silexan

^b^Positive values favour Silexan

**Fig. 3:**
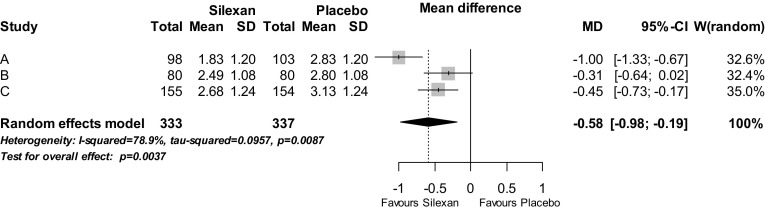
Clinical Global Impressions, item 2, change from project admission—assessment at treatment end (*SD* standard deviation, *MD* mean value difference, *CI* confidence interval, *W* weight)

Figure 3 shows the meta-analysis results for CGI item 2 (global change between project admission and treatment end) which were similar to those for item 1. In addition to the over-all significant mean value difference (*p* = 0.004), two out of the three trials also showed a significant treatment group difference favouring Silexan. The standardised mean value difference (Hedges’ G) was 0.49 (95% CI 0.17; 0.81, *p* = 0.003).

### Response and remission

**Fig. 4:**
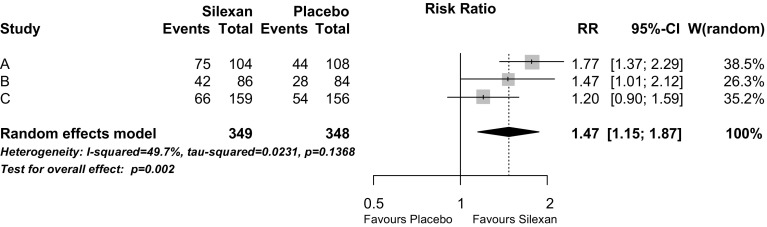
Analysis of response—number of patients with a Hamilton Anxiety Rating Scale total score reduction by at least 50% between baseline and treatment end (*RR* risk ratio, *CI* confidence interval, *W* weight)

The percentage of patients whose HAMA total score decreased by at least 50% of the baseline value during randomised treatment was significantly larger in participants treated with Silexan as compared to those who received placebo (Fig. 4; *p* = 0.002). The corresponding number needed to treat (NNT) derived from the meta-analysis was 6. When response was defined by a score of 1 or 2 for CGI item 2 (i.e., much or very much improved), a risk ratio of 1.69 (95% CI 1.44; 2.00, *p* < 0.001) favouring Silexan was determined by meta-analysis, with individual study risk ratios ranging between 1.53 and 1.84 (meta-analysis NNT 5).

**Fig. 5:**
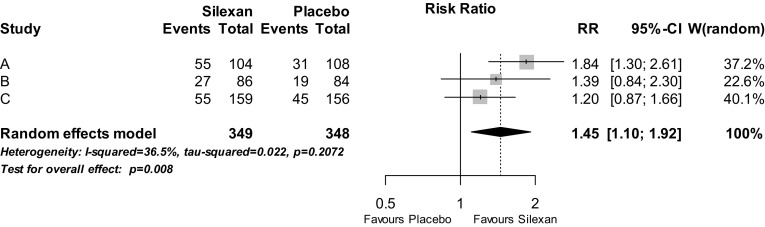
Analysis of remission—number of patients with a Hamilton Anxiety Rating Scale total score < 10 points at treatment end (*RR* risk ratio, *CI* confidence interval, *W* weight)

Remission, defined by a HAMA total score of less than ten points at treatment end, was also significantly more frequent in the Silexan group as compared to placebo (*p* = 0.008; Fig. 5; meta-analysis NNT 8). When the threshold for remission was tightened from less than ten to less than or equal to seven points, two out of the three trials still showed advantages for Silexan, and the meta-analysis model resulted in a pooled risk ratio of 1.37 (95% CI 1.05; 1.79, *p* = 0.022) favouring Silexan.

### Effect on disturbed sleep related to SSAD

According to HAMA item ‘Insomnia’ (defined by difficulty in falling asleep, broken sleep, unsatisfying sleep, fatigue on waking, dreams, nightmares, and night terrors) 10 weeks’ administration of Silexan led to a significantly more pronounced symptom reduction than placebo treatment (Table 4). More detailed insight into the effect of the investigational treatments on sleep was obtained by means of the PSQI which was administered in studies A and B. The meta-analysis of total score of the scale showed significantly more pronounced symptom alleviation in the Silexan group as compared to placebo (Table 5). Advantages for Silexan were also observed in the meta-analyses for PSQI components Sleep quality (*p* = 0.051), Sleep latency (*p* < 0.001), Sleep disturbances (*p* = 0.014), and Daytime dysfunction (*p* < 0.001), whereas the remaining components showed no significant differences between Silexan and placebo.

### Health-related quality of life

Health-related quality of life was assessed in studies A and C using the SF-36 health status questionnaire. The pooled meta-analysis mean value differences for the mental and physical health subscores of the SF-36 are presented in Table 5. Both scales showed significant treatment group differences favouring Silexan. Moreover, for both domains, superiority of Silexan over placebo was also observed in each study evaluated independently.

### Safety

**Fig. 6:**
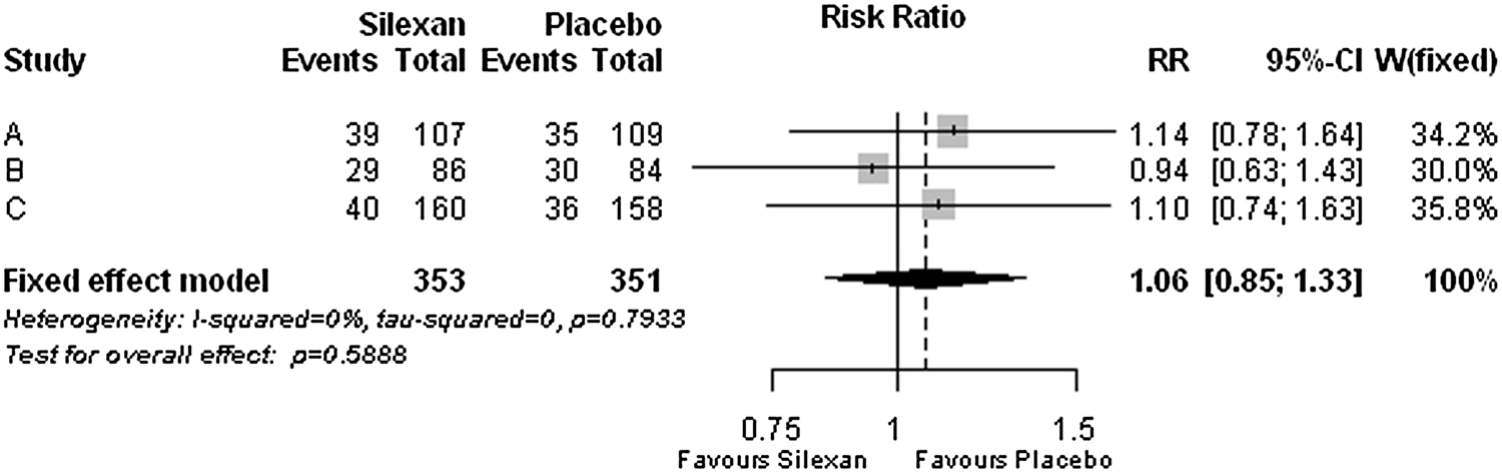
Number of patients with any adverse events (*RR* risk ratio, *CI* confidence interval, *W* weight)

For Silexan and placebo similar percentages of patients with any AEs during randomised treatment were observed for all included trials, with two trials slightly favouring placebo and one trial slightly favouring Silexan (*p* = 0.589; Fig. 6) and a meta-analysis number needed to harm (NNH) of 55.

Serious AEs were reported in two patients in each treatment group of trial A and in one patient in the Silexan group as well as in two patients in the placebo group of trial C, whereas there were no serious events in trial B (meta-analysis risk ratio: 0.75 favouring Silexan, 95% CI 0.17; 3.33, *p* = 0.710).

Across all trials, a total of six out of 356 patients randomised to Silexan and four out of 353 randomised to placebo were withdrawn due to an AE (meta-analysis risk ratio: 1.43 favouring placebo, 95% CI 0.43; 4.76, *p* = 0.557; NNH 182). AEs leading to premature withdrawal in Silexan-treated patients were female genital pain (not related), two cases of nausea (one not related, one possibly related), gastritis (unlikely related), eructation (probably related), and oral discomfort (possibly related). In the placebo group patients were withdrawn for subileus, anorexia, nausea and dizziness, as well as for diverticular perforation.

## Discussion

Subthreshold anxiety is not only a diagnostic entity in its own right, but also an important feature or co-morbidity symptom of several psychiatric (most notably depression) and somatic diseases. Our meta-analysis demonstrates that Silexan is superior to placebo in reducing the anxiety-associated symptoms in patients suffering from SSAD, including MADD or restlessness and agitation disorder. Significant superiority of Silexan was observed for psychic as well as for somatic manifestations of anxiety. While no empirically derived minimal clinically important difference (MCID) has yet been published for HAMA total score change or for the difference between drug and placebo, the protocols of the included trials have assumed a treatment group mean value difference in HAMA total score reduction of 2.5 or three points to be clinically important. The 3.8-point difference observed in our meta-analysis actually exceeds this margin. The clinical relevance of the observed effect is also supported by significant superiority of Silexan over placebo regarding the percentage of patients who showed an at least 50% reduction of their baseline HAMA total score, or who had a rating of one or two points for CGI item 2 (i.e., very much or much improved) at treatment end, criteria that are commonly used in pharmacotherapy studies as indicators of an important treatment effect [32].

The effect size point estimates of 0.45 for HAMA total score change and of 0.49 for CGI global improvement observed in this trial are not easily compared to the literature because published data on treatment effect sizes in SSAD are sparse. For GAD, Hidalgo and colleagues have published a review of 21 double-blind, placebo-controlled trials which shows effect sizes of 0.50 for pregabalin, 0.45 for hydroxyzine, 0.42 for venlafaxine, 0.38 for all benzodiazepines (alprazolam, diazepam, lorazepam), 0.36 for all SSRIs (paroxetine, sertraline, fluvoxamine, escitalopram), and 0.17 for buspirone for HAMA total score change [33]. For Silexan, effect sizes of 0.37 and of 0.50 have been reported in GAD for dosages 80 and 160 mg/day, respectively [13]. Moreover, the NNT of six patients exposed to observe one additional treatment responder defined by a ≥ 50% decrease of the HAMA total score was comparable to that determined for second-generation antipsychotics (NNT: 6) [34] and lower than that observed in a recent study on vilazodone (NNT: 10) [35] in patients with GAD (no NNTs could be found in the literature for patients with SSAD). NNTs published for remission [35–37] were mainly based on criteria substantially different from our prospectively defined criteria and were thus not comparable. Depping and colleagues [34] determined an NNT of ten for quetiapine when remission was defined by a HAMA total score < 17 points, compared to an NNT of eight in our meta-analysis for Silexan although we required a HAMA total score of less than 10 points for remission. These results suggest that the therapeutic effect of Silexan 80 mg/day in SSAD may likely be within the range of that of synthetic anxiolytic drugs that are recommended as first-line treatments in anxiety disorders [38].

It is important to note that the anxiolytic effect of Silexan was evident both in the observer ratings as well as in the patients’ anxiety self-ratings. Patient relevance is supported by significant superiority of the product over placebo regarding health-related quality of life, where a beneficial drug effect was again observed for both mental and physical aspects of disease-associated impairment.

Disturbed sleep has been recognised as an important co-morbidity of anxiety [39]. Our results indicated that Silexan has a beneficial effect on anxiety-related sleep impairments. Detailed results of trials A and B published elsewhere indicate a clear association between the alleviation of anxiety symptoms and the improvement of sleep, with a slight delay in PSQI total score decrease as compared to that of the HAMA [14, 16]. These results indicate that the improvement of sleep is secondary to the anxiolytic effect of Silexan. Importantly, while the product improves the quality and shortens the latency of sleep, it also has a favourable effect on sleep-related daytime dysfunction. The observation is consistent with previous research according to which Silexan has a calming but not a sedating effect [24, 40].

Our literature search showed that information regarding the anxiolytic efficacy of Silexan in patients with subsyndromal anxiety is sparse as the three trials included into our meta-analysis are the only ones performed in this patient population to date. Moreover, only one of the trials investigated patients with ‘pure’ SSAD, i.e., without relevant psychiatric co-morbidity. While this imposes certain limitations regarding generalisability, our results are nevertheless based on the whole body of evidence that pertains to the efficacy of Silexan in subthreshold anxiety.

Sedation has been described for many of the drugs currently recommended as first-line treatments of anxiety disorders, including selective serotonin reuptake inhibitors (SSRIs) and the antihistamine hydroxyzine [38]. Unwanted sedation may cause significant limitations in a patient’s ability to pursue essential activities of daily living, e.g., to operate machinery or to drive a vehicle. Other common side effects of SSRIs and/or hydroxyzine include anticholinergic reactions, gastrointestinal disturbances, weight gain, sexual dysfunction, or agitation and irritability [41, 42]. For hydroxyzine the European Medicines Agency’s Pharmacovigilance Risk Assessment Committee has recently issued a warning according to which the drug may cause serious cardiac side effects including QT interval prolongation and torsades de pointes [43]. Anxiolytic drugs may thus cause disturbing, partly serious side effects, which may be one of the reasons for the undertreatment of subthreshold anxiety [44]. Our meta-analysis shows that the rates of serious and non-serious adverse events observed during 10 weeks’ treatment with Silexan differed hardly from those reported for patients exposed to placebo. Indeed, reports published for Silexan to date suggest that eructation and dyspeptic symptoms as well as allergic skin reactions may be the only specific adverse effects associated with the product [24]. Patients may perceive the absence of side effects that are likely to interfere with essential aspects of daily living as an important contribution to their quality of life. This interpretation is supported by the marked improvements observed in the SF-36 quality of life questionnaire in patients treated with Silexan.

Lavender oil is a complex, multi-ingredient mixture in which more than 160 different substances have been identified [24]. The anxiolytic properties of the drug have been ascribed to different ingredients, among them linalool and linalyl acetate [45]. Marketed oils from lavender differ greatly with regard to quality linalool or linalyl acetate content [46]. It is, therefore, important to note that the results presented in this paper apply to Silexan but not to other lavender oil containing products.

In conclusion, our meta-analysis shows that Silexan has a statistically significant and clinically meaningful anxiolytic effect in subthreshold anxiety. The product had a beneficial effect on disturbed sleep secondary to anxiety and was associated with improvements in health-related quality of life. Silexan was well tolerated, with adverse event rates similar to those observed for placebo.
